# Impact of Adenomyosis on Infertile Patients—Therapy Options and Reproductive Outcomes

**DOI:** 10.3390/biomedicines10123245

**Published:** 2022-12-13

**Authors:** Veronika Günther, Leila Allahqoli, Georgios Gitas, Nicolai Maass, Karolin Tesch, Johannes Ackermann, Paula Rosam, Liselotte Mettler, Sören von Otte, Ibrahim Alkatout

**Affiliations:** 1 Department of Obstetrics and Gynecology, University Hospitals Schleswig-Holstein, Campus Kiel, Arnold-Heller-Strasse 3 (House C), 24105 Kiel, Germany; 2 University Fertility Center, Ambulanzzentrum des UKSH gGmbH, Arnold-Heller-Strasse 3 (House C), 24105 Kiel, Germany; 3 School of Public Health, Iran University of Medical Sciences (IUMS), Tehran 14167-53955, Iran; 4 Private Gynecologic Practice, Chrisostomou Smirnis 11Β, 54622 Thessaloniki, Greece; 5 Department of Radiology and Neuroradiology, University Hospitals Schleswig-Holstein, Campus Kiel, Arnold-Heller-Strasse 3 (House C), 24105 Kiel, Germany

**Keywords:** adenomyosis, infertility, diagnosis, treatment options, reproductive outcome

## Abstract

Adenomyosis is associated with a negative impact on reproductive outcomes. Although adenomyosis is detected more frequently in women of late reproductive age, its impact on pregnancy rates is important because, in today’s world, family planning has shifted towards the late reproductive phase of life for many women. Although the diagnostic indications for imaging studies are well-known, we lack strict diagnostic criteria and classification systems concerning the extent of the disease. Selecting the optimal evidence-based treatment option for adenomyosis is difficult because of the paucity of evidence concerning the association between fertility and the degree and composition of adenomyosis. Furthermore, the treatment of infertility might interfere with the treatment of adenomyosis due to the presence of pain. The aim of this review is to analyze the association between adenomyosis and infertility, and describe treatment options to enhance reproductive outcomes. The following aspects will be addressed in detail: (a) prevalence and causes of adenomyosis, (b) diagnostic tools with imaging techniques, (c) clinical symptoms, (d) proposed pathomechanism of adenomyosis and infertility, and (e) different treatment approaches (pharmacological, surgical, others) and their impact on reproductive outcomes.

## 1. Introduction

Adenomyosis, also known as endometriosis genitalis interna, is a special form of endometriosis in which endometrial epithelial cells and stromal fibroblasts invade the uterine myometrium. This leads to reactive fibrosis, hyperplasia and hypertrophy of the surrounding smooth muscle cells, as well as severe menstrual and inter-menstrual bleeding and recurrent pain [1].

The prevalence of adenomyosis has been reported to range from 5% to 70%. Women younger than 40 years of age appear to be affected in approximately 20% of the cases, and women between the ages of 40 and 50 years in 80% [2]. However, it is difficult to quantify the incidence precisely because we lack a uniform definition, diagnostic criteria based on noninvasive tests, or even laparoscopic criteria for the diagnosis [3].

## 2. Pathogenesis and Risk Factors

A number of theories have been proposed for the pathogenesis of adenomyosis [4]:

- Myometrial invasion of the endometrium
- De novo development from embryonic remnants of the Müllerian ducts
- Invagination of the stratum basalis of the endometrium along the lymphatic vessels of the myometrium
- Adenomyosis from bone marrow stem cells
- Archimetrosis, a novel theory concerning the pathogenesis of uterine adenomyosis and endometriosis, which is connected with the evolution of the stratum vasculare, tissue injury and repair. Hypercontractility seems to represent a risk factor [5].

Until recently, a high risk of adenomyosis was linked to a large number of births, spontaneous or induced abortion, and endometrial hyperplasia. Currently, adenomyosis is diagnosed with increasing frequency in infertile patients, which might be due to better imaging techniques. Other risk factors include endometriosis, smoking, and surgical trauma, such as cesarean section or curettage [6]. Interestingly, adenomyosis has been reported in 60% of postmenopausal women on prolonged tamoxifen therapy. Thus, the disease appears to be estrogen-dependent and can be reactivated post-menopause in cases of preexisting lesions [7].

## 3. Clinical Symptoms

Adenomyosis is most frequently diagnosed in the fourth or fifth decade of life, on the basis of a bleeding disorder and menstrual pain. The predominant symptoms are hyper- and dysmenorrhea, which occur in about 50% and 25% of women, respectively [1,8]. Additionally, adenomyosis seems to be a risk factor for infertility, but due to a small number of case series with low level of evidence, it is difficult to mention exact data [9]. Those studies report differences of adenomyosis prevalence in infertile patients, ranging from 7 to 28 % [10,11]. Puente et al. and Sharma et al. published data concerning an adenomyosis rate of 24.4% in infertile women, especially in those who suffered from recurrent miscarriages and recurrent implantation failures, in older women seeking IVF treatment, and in those with endometriosis [12,13]. To investigate the relationship between endometriosis and adenomyosis, Bourdon et al. established an interesting observational cohort study: the pregnancy outcome in women with adenomyosis and endometriosis lesions (study group, n = 214) was compared with the pregnancy outcome in women with only adenomyosis lesions (control group, n = 53). The miscarriage rate was significantly higher among women with adenomyosis and endometriosis lesions compared with women in the control group (61/214 (28.5%) versus 6/53 (11.3%), respectively, *p* = 0.009). A deep infiltrating endometriosis increased this risk significantly [14]. Clinical symptoms, concomitant diseases of adenomyosis and their frequencies are summarized in Table 1.

Bleeding disorders may be related to the expanded surface of the endometrium in the enlarged uterus. Dysmenorrhea and dyspareunia can be explained by myometrial hypercontractility and the increased contractile amplitude of the uterine smooth muscle cells [15]. This hypercontractility favors infertility: adenomyosis has a negative impact on implantation rates during in vitro fertilization, pregnancy and live birth rates in general, and raises the risk of miscarriage [17,18]. Furthermore, adenomyosis is associated with higher rates of obstetric complications, such as preterm birth and preterm rupture of the amniotic membranes [19,20].

## 4. Diagnosis of Adenomyosis

Previously, adenomyosis was diagnosed in premenopausal women solely on the basis of pathological examination after hysterectomy [21,22]. The first imaging tool employed to diagnose adenomyosis was hysterosalpingography [23]. The latter investigation is no longer used because of its poor overall accuracy. Today, the diagnosis can be established by a transvaginal ultrasound (TVS) scan or magnetic resonance imaging (MRI) [24]. The former procedure is the primary diagnostic tool [25]. The two types of adenomyosis, diffuse and focal, can be distinguished in a transvaginal ultrasound scan. Adenomyoma is a special form of focal adenoymosis. Table 2 summarizes the features of diffuse and focal disease.

**Figure 1:**
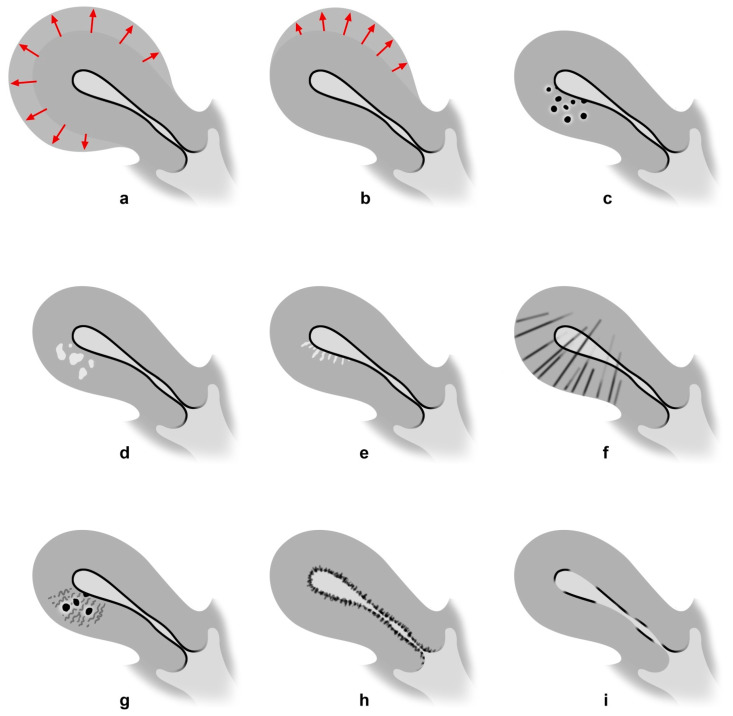
Sonographic findings in adenomyosis [30]. (**a**) Spherical, (**b**) wall difference in favor of the anterior or posterior wall, (**c**) subendometrial cysts, (**d**) echogenic islands, (**e**) fan-shaped shadowing, (**f**) echogenic subendometrial lines or buds, (**g**) translesional vascularity, (**h**) irregular junctional zone, (**i**) interrupted junctional zone.

Transvaginal ultrasound signs of adenomyosis include a spherical enlargement of the uterus with an asymmetric thickening of the anterior or posterior wall. As a characteristic sonographic sign associated with the presence of adenomyosis, the so-called question mark sign or question mark form can be described: it is a special form of the uterus, wherein the cervix is directed anteriorly towards the uterine bladder, followed by a uterine corpus flexed backward. This leads to the typical endometrial shape, like a question mark. In the presence of this formation, the diagnosis of adenomyosis can be established with a sensitivity of 92% and a specificity of 75% [2]. Further ultrasound signs are heterogeneous and hypoechoic, poorly described areas in the myometrium, with or without anechoic lacunae or cysts of varying size, as well as linear striation radiating from the endometrium into the myometrium with a poorly defined junctional zone (JZ) [27]. Adenomyosis can be diagnosed with substantial certainty when three or more of these sonographic criteria are fulfilled [28,29]. Figure 1 is a schematic diagram of sonographic findings in accordance with the Morphological Uterus Sonographic Assessment (MUSA) group [27,30]. Furthermore, adenomyosis may involve between one and three of the uterine layers, which seems to correlate with clinical symptoms: 1. the JZ (the inner myometrium, also called the subendometrial layer); 2. the middle myometrium (the myometrium between the vascular arcade and the JZ); and 3. the outer myometrium (the subserosal layer, i.e., the layer between the serosa and the vascular arcade).

Three-dimensional transvaginal ultrasound reveals the following features: JZ of at least 8 mm with additional irregularities, asymmetry in the myometrium, and hypoechoic striations, such as intramural fibroids. When at least two of the above-mentioned ultrasound features are present, the accuracy is 90% (sensitivity, 92%; specificity, 83%; positive predictive value, 99%; and negative predictive value, 71%) [31].

MRI of the uterus is a second-line examination for the diagnosis of adenomyosis, especially in cases of inconclusive ultrasound evaluation or as a preoperative measure prior to surgical intervention. An MRI can also distinguish between subtypes of adenomyosis, and more precisely differentiate between adenomyosis and fibroids than TVS [32,33]. The JZ shows fluctuations in thickness depending on hormone levels. Thus, the late proliferative phase appears to be the optimal time for the examination. Physiological contractions may cause the JZ to appear focally disseminated and this should not be confused with adenomyosis-related changes [34].

Both MRI and TVS appear to be equally capable of making the diagnosis of adenomyosis, but MRI seems to be superior to TVS in identifying adenomyosis. It has equal sensitivity but a higher specificity than TVS (sensitivity: MRI 0.70 (0.46–0.87), TVS 0.68 (0.44–0.86) (*p* = 0.66); specificity: MRI 0.86 (0.76–0.93), TVS 0.65 (0.50–0.77) (*p* = 0.03)) [29,35]. Measurement of the JZ serves a further diagnostic tool. Adenomyosis may be considered likely when the thickness of the JZ exceeds 12 mm, although there is no clear definable JZ on MRI in around 20% of premenopausal women [36]. Adenomyosis can also be suspected when the thickness of the JZ is between 8 mm and 12 mm or in the presence of other specific signs, such as a relative thickening of the JZ in a localized area, a poor definition of the JZ margins, or high-signal foci on T2- or T1-weighted sequences [23,35].

Adenomyosis is not only a pathology of adults. Adolescents can also be affected, suffering from mild to moderate typical painful symptoms, and dysmenorrhea seems to be the most commonly reported symptom [37]. Exacoustos et al. performed an observational study on this topic and described the following sonographic findings in case of adenomyosis in adolescents, especially affecting the posterior uterine wall (58%) and the outer myometrial layer (93%): myometrial hyperechoic areas, uterine wall asymmetry, intramyometrial cystic areas, and some types of junctional zone alterations were noticed. Interestingly, adolescents with diffuse adenomyosis were significantly older and showed a high percentage of heavy menstrual bleeding compared with those suffering from focal adenomyosis [37].

## 5. Effect of Adenomyosis on Reproductive Outcomes

**Figure 2:**
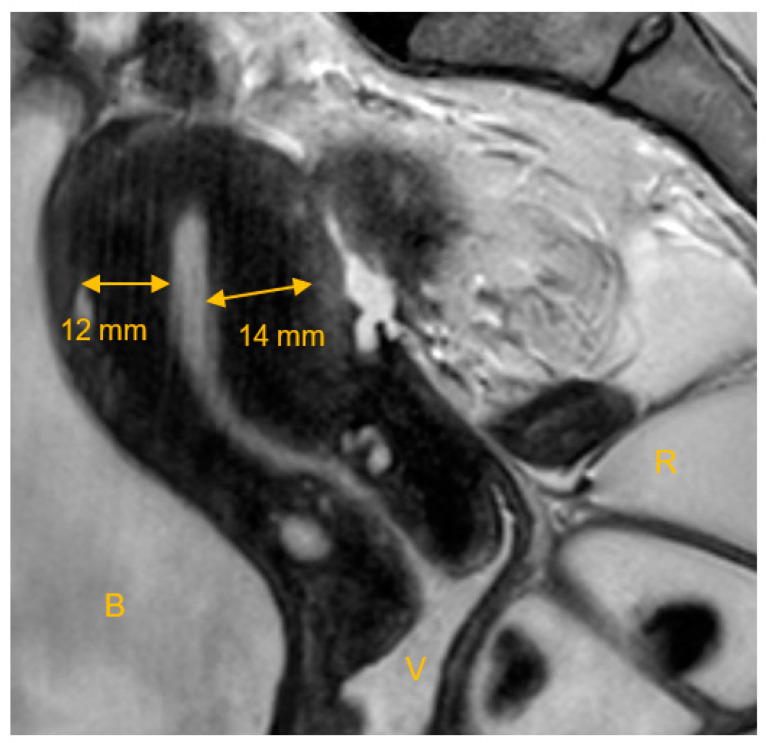
MRI characteristics of the uterine junctional zone. T2-weighted MRI in the sagittal plane, showing the widened junctional zone marked with arrows. B: bladder, V: vagina filled with ultrasound gel, R: rectum filled with ultrasound gel. Image acquisition on 3T, Ingenia Philips Healthcare, with a T2 TSE sequence.

The junctional zone (JZ) is of crucial importance in the evaluation of adenomyosis and its impact on fertility. In 1983, Hricak et al. first described the functional uterine zone, which is the junction between the endometrium and the inner myometrium [38]. There are three layers that can be distinguished on T2-weighted images: (1) an area of high signal intensity corresponding to the endometrial stripe; (2) an inner area of low signal intensity close to the basal endometrium, the JZ or the subendometrial layer; and (3) an outer region of moderate signal intensity, which is the subserosal zone or the outer myometrium [35,39]. Figure 2 shows MRI characteristics of the JZ.

A variety of morphologies may be present in adenomyosis, ranging from slight localized expansion of the JZ to massive myometrial hyperplasia and fibrosis, which might influence the tonus of the uterus and the contours of the endometrial cavity in general [12]. Hyperactivity of the myometrium is often observed in patients with adenomyosis. Especially at the cellular level, changes in myocytes can be found, leading to a disrupted calcium circulation, which implies irregular muscle contractions, dysfunctional uterine hyperperistalsis with increased intrauterine pressure, and the development of hyperplastic myometrial tissue [35].

**Figure 3:**
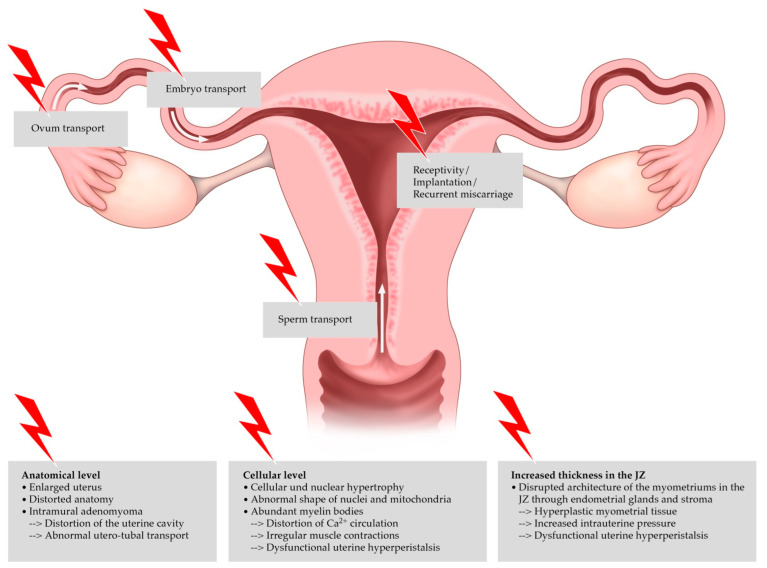
Negative impact of adenomyosis on reproductive outcomes. Adenomyosis leads to changes in the junctional zone (JZ), the anatomy of the uterus itself, and at the cellular level. The transport of the sperm, egg, and finally the embryo is complicated by dysfunctional peristalsis.

The impact on peristalsis, uterine contraction, and fertility may differ in accordance with various morphologic changes, but there is no consensual classification system for the extent of disease based on image morphology [12]. The transport of sperms and the embryo, as well as endometrial function and its receptivity, are affected by muscular peristalsis in the JZ, and consequently play an important role in implantation [17,40]. Figure 3 shows the different locations in the female genital tract and the negative impact of adenomyosis on the individual steps of reproduction.

**Figure 4:**
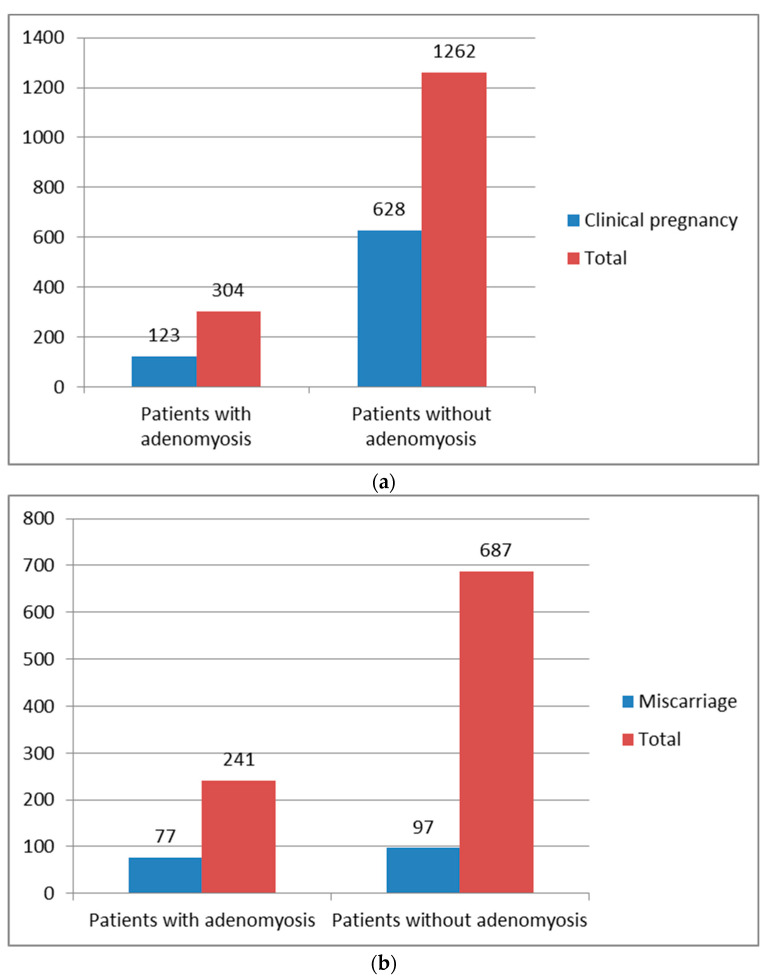
(**a**): Clinical pregnancy rates after ART in patients with and without adenomyosis [43]. The clinical pregnancy rate achieved after ART was 123/304 (40.5%) in women with adenomyosis versus 628/1262 (49.8%) in those without adenomyosis. (**b**): Miscarriage rates after ART in patients with and without adenomyosis [43]. A miscarriage was observed in 77/241 women with adenomyosis (31.9%) and in 97/687 in those without adenomyosis (14.1%).

Adenomyosis is associated with advancing maternal age, which in turn is correlated with lower pregnancy rates due to reduced oocyte quality and lower ovarian reserve [41]. Rasmussen et al. analyzed reproductive outcomes in relation to the thickness of the JZ. Fertile women appear to have a median JZ width of 5.2 mm, and a mere 12% of women had a JZ of 8–12 mm [42]. In cases of recurrent miscarriage and repeated implantation failure after assisted reproductive technologies (ART), a thicker JZ was seen on 3D-TVS (38% and 35%, respectively) [12]. A thickened and irregular JZ (maximum > 10 mm on MRI) has been frequently observed in cases of peritoneal endometriosis detected by laparoscopy. The frequency varies between 27% and 79%. Thus, adenomyosis appears to be present in about one third of women with surgically treated endometriosis [8]. Vercellini et al. performed a meta-analysis in 2014 and analyzed the effect of adenomyosis on the outcome of ART. The clinical pregnancy rate after ART was 123/304 (40.5%) in women with adenomyosis versus 628/1262 (49.8%) in those without adenomyosis. The RR of clinical pregnancy ranged from 0.37 (95% CI, 0.15–0.92) to 1.20 (95% CI, 0.58–2.45), with a significant heterogeneity among studies (*p* = 0.023) that can be caused by methodological, clinical or statistical factors. Due to this heterogeneity, the results should be interpreted with caution. A miscarriage was observed in 77/241 women with adenomyosis (31.9%) and in 97/687 women without adenomyosis (14.1%). The RR of miscarriage ranged from 0.57 (95% CI, 0.15–2.17) to 18.00 (95% CI, 4.08–79.47), *p* = 0.005 [43]. Figure 4a,b shows clinical pregnancy and miscarriage rates.

## 6. Treatment Options in Patients with Adenomyosis

Analogous to the treatment of endometriosis, the treatment of adenomyosis is based on individual symptoms and family planning. Basically, a distinction is made between pharmacological, surgical and other treatment approaches [18,44]. The ESHRE guideline for endometriosis and infertility does not recommend any treatment leading to ovarian suppression after surgery and/or before ART with the aim of improving fertility [45]. Unfortunately, such a detailed guideline does not exist for adenomyosis. Table 3 shows the different therapy options for patients with adenomyosis.

## 7. Reproductive Outcomes after the Treatment of Adenomyosis

### 7.1. Pharmacological Treatment Options

Nonsteroidal anti-inflammatory drugs (NSAIDs) are most often used as a first-line treatment for women with pain and endometriosis. The mechanism of action is based on blocking the prostaglandin production through the inhibition of cyclooxygenase, an enzyme responsible for the formation of prostaglandins. Common NSAIDs (aspirin^®^, naproxen, ibuprofen) are very effective in reliving dysmenorrhea [46]. However, we have just a few randomized trials on the use of these agents in endometriosis and none of the studies were performed in patients with adenomyosis [47]. NSAIDs are associated with a negative impact on fertility because they may delay ovarian follicle rupture. Nevertheless, there is some evidence that NSAIDs may be used as co-treatment in the ART procedure due to the following theory: adenomyosis may lead to a local hyper-production of uterine prostaglandins, increased uterine tonus, and high-amplitude contractions, thus reducing the possibility of an IVF cycle with successful embryo implantation. It should be noted that NSAIDs have been used to inhibit the negative prostaglandin effect [48].

Combined oral contraceptives (OCs) act by suppressing ovulation and consequently hindering endometrial proliferation. Patients with dysmenorrhea and bleeding disorders, such as hypermenorrhea or meno- or metrorrhagia, benefit especially from this approach. In the extended cycle regimen, OC can be taken for three or six consecutive months before abortion bleeding is induced [49]. In two thirds of women with symptomatic endometriosis or adenomyosis, the use of OCs provides satisfactory pain control in the long term. However, we have almost no data concerning the impact of OCs on the subsequent improvement of fertility [50].

Progestogen mono (Progestin-only pill, POP) at the ovulation-inhibiting dose leads to suppression of ovulation and subsequent endometrial atrophy. The benefits of this therapy are good pain and bleeding control in patients with dysmenorrhea or bleeding disorders. The disadvantages of POP are bleeding disorders such as spotting, and depressive moods especially at the beginning of use.

Visanne^®^ (Dienogest (DNG) 2 mg) has been in the market since 2010 and is officially approved for the treatment of endometriosis. DNG is a progestogen with a pronounced effect on the endometrium. It is well-suited for cycle stabilization and has long been used in gynecology for hormonal contraception and hormone replacement therapy. DNG acts in endometriosis by reducing the endogenous production of estradiol, thus suppressing the trophic effects of estradiol in the eutopic as well as ectopic endometrium. With continuous administration, DNG induces a hypoestrogenic, hypergestagenic endocrine state that causes initial decidualization of endometrial tissue, in which glycogen and fat are stored and cells become rounded and tightly packed, followed by atrophy of endometriotic lesions. The antiproliferative effect on the endometrium, combined with potent secretory-transformative and anti-inflammatory activity, make DNG an ideal candidate for the treatment of endometriosis. Visanne^®^ significantly reduces menstrual bleeding, dysmenorrhea, premenstrual pain, dyspareunia, and pelvic pain. In patients with adenomyosis, DNG might cause bleeding disorders, especially menometrorrhagia [51]. Women must be informed that ovulation is suppressed by regular intake of Visanne^®^, but the agent is not officially approved for contraception [52].

A small number of studies with limited sample sizes have analyzed reproductive outcomes after a Progestogen mono pretreatment for endometriosis [53]. Barra et al. performed a retrospective analysis including 151 women who had failed a previous IVF cycle and all subsequent embryo transfers, and had endometriosis diagnosed by imaging studies. The treatment group comprised 63 women who received 2 m DNG daily for three months, while 88 women underwent the next IVF cycle without any previous hormonal treatment. The rates concerning cumulative implantation, clinical pregnancy, and live birth were significantly higher in the DNG-treated group (39.7%, 33.3% and 28.6%) than in the non-treated group (23.9%, 18.2% and 14.8%; *p* = 0.049, 0.037 and 0.043, respectively). Additionally, the use of DNG significantly increased the number of retrieved oocytes (*p* = 0.031), two-pronuclear embryos (*p* = 0.039) and blastocysts (*p* = 0.005) in women with endometriomas of a diameter ≥ 4 cm. The authors conclude that pretreatment with DNG leads to better reproductive outcomes [54].

Another group came to similar conclusions in a study comprising 38 patients treated with DNG, 70 patients pretreated with GnRH analogs, and a further 70 control patients who received no hormonal therapy for 6 months preceding IVF. All of the women had undergone laparoscopic surgery for ovarian endometriomas previously. Women who received DNG pretreatment had a 2.5-fold higher clinical pregnancy rate (44.7% versus 16.7%, *p* = 0.012), and a three-fold higher delivery rate (36.8% versus 11.1%, *p* = 0.013) than controls [55].

In a third study, also with relatively small sample sizes (n = 33 in the DNG group and n = 35 in the control group), the authors analyzed reproductive outcomes after 12 weeks of pretreatment with DNG vs. no hormonal pretreatment [56]. The numbers of growing follicles, retrieved and fertilized oocytes and blastocysts were significantly lower in the DNG group than in controls. Although there was no significant difference in implantation rates between groups, the cumulative pregnancy rate and live birth rate were lower in the DNG group than in controls [56]. It is important to state, that all the above cited studies address reproductive outcome after progesterone mono in endometriosis. They do not explicitly include patients with adenomyosis or exclude patients with endometriosis genitalis externa. Further studies analyzing the reproductive outcome in patients with adenomyosis are needed.

Levonorgestrel intrauterine system (LNG-IUS) does not cause ovarian suppression, but counteracts dysmenorrhea and bleeding disorders thorugh (a) the impact of progestogen on adenomyosis foci, (b) atrophy of the endometrium, and (c) control of endometrial factors that changed during adenomyosis, such as reduced expression of growth factors and their receptors associated with hypermenorrhea [35,57]. The LNG-IUS is an approved therapy option for women with adenomyosis and completed family planning. Nevertheless, some studies have addressed reproductive outcomes after pretreatment with LNG-IUS following IVF. Liang et al. studied 358 women with adenomyosis undergoing IVF, 134 of whom were enrolled in the LNG-IUS group and 224 in the control group [58]. The authors noted higher rates of implantation (32.1% vs 22.1%, *p* = 0.005), clinical pregnancy (44% versus 33.5%, *p* = 0.045), and ongoing pregnancy (41.8% vs 29.5%, *p* = 0.017) in the LNG-IUS group compared to the control group [58]. One explanation for these positive results might be the influence of LNG-IUS on adenomyosis, especially on the expression levels of steroid receptor coregulators, such as transcriptional intermediary factor 2 or nuclear receptor corepressor. These coregulators were reduced after treatment with LNG-IUS [59].

Gonadotropin-releasing hormone analogs (GnRH-a) are the best studied of all pharmacological treatment options for adenomyosis and subsequent infertility. The effect of GnRH analogs proceeds in two phases: first, there is a so-called flare-up effect. Primarily, the stimulation and release of FSH and LH initially increases estrogen biosynthesis and secretion. Secondarily, persistent binding to the GnRH receptor causes a downregulation of FSH and LH, which leads to the suppression of ovarian estrogen biosynthesis and secretion. Two routes of administration may be used: depot preparations are applied subcutaneously or through the intramuscular route, and regular application consists of a nasal spray. After about 4 weeks, average estradiol levels around 20 pg/ ml are measured after depot administration, which corresponds to the postmenopausal serum estradiol concentration. Nasal application also causes a drop in estrogen after the initial flare-up effect, but not as extensively as a depot preparation. Average estradiol levels of 40 pg/mL are measured after 4–8 weeks, and 30 pg/mL after 24 weeks [60,61]. The use of GnRH analogs for symptomatic adenomyosis has decreased considerably over the past years due to the availability of better tolerated alternatives (see above). Osteoporosis, hot flashes, vaginal atrophy, and depressive moods are among the most common side effects. Due to concomitant osteoporosis, the duration of treatment with GnRH agonists without add-back therapy should not exceed 3 months. In order to enhance reproductive outcomes after ART, GnRH analogs may be used for 3 to 6 months as a means of downregulation. Given that the GnRH receptor is also found in adenomyotic lesions, and GnRH analogs have a direct antiproliferative effect within the myometrium, the treatment reduces the inflammatory response and angiogenesis and also induces apoptosis in the tissue [62]. This might exert a favorable effect on implantation. Long-term treatment with GnRH agonists or an ultra-long protocol may have a therapeutic effect on adenomyosis and improve the outcome of ART [63].

Hou et al. performed an observational cohort study comprising three groups: (a) 362 patients with adenomyosis using the ultra-long GnRH agonist protocol, (b) 127 patients with adenomyosis using the long GnRH agonist protocol, and (c) 3471 patients with tubal infertility using the long GnRH agonist protocol [64]. Long GnRH agonist treatment reduced the clinical pregnancy rate (OR 0.492, 95% CI 0.327 to 0.742, *p* < 0.001), implantation rate (OR 0.527, 95% CI 0.350 to 0.794, *p* = 0.002), and live birth rate (OR 0.442, 95% CI 0.291 to 0.673, *p* < 0.001), and increased the miscarriage rate (OR 3.078, 95% CI 1.593 to 5.948, *p* < 0.001) in adenomyosis patients (Group b) compared to those with tubal infertility (group c). Ultra-long GnRH agonist treatment in patients with adenomyosis (Group A) increased the clinical pregnancy rate (OR 1.925, 95% CI 1.137 to 3.250, *p* = 0.015), implantation rate (OR 1.694, 95% CI 1.006 to 2.854, *p* = 0.047) and live birth rate (OR 1.704, 95% CI 1.012 to 2.859, *p* = 0.044) compared to long GnRH agonist treatment (Group b) [64]. The authors conclude that adenomyosis has a negative impact on IVF outcomes, and that the ultra-long GnRH agonist protocol provides a better reproductive outcome in those patients [64].

Younes et al. reported similar results in their meta-analysis evaluating the effects of adenomyosis on in vitro fertilization. Whereas adenomyosis has a negative impact on implantation, clinical pregnancy and live birth rate, pretreatment with GnRHa increases pregnancy rates [65].

In contrast to the majority of studies on this subject, a systematic review and meta-analysis performed by Cozzolino et al. yielded different results [66]. The authors analyzed pregnancy outcomes in patients with untreated adenomyosis and surgically or medically treated adenomyosis. After surgery, the authors observed an increased natural conception rate in women with adenomyosis. In contrast, the treatment with GnRHa did not lead to better IVF outcomes. Only three studies concerning this topic were included in the meta-analysis [66]. The authors state that most of the studies did not make a distinction between focal and diffuse adenomyosis; such a distinction might have yielded different results in terms of clinical presentation and treatment options [66].

New findings concerning pathogenic mechanisms have led to new medical approaches for the treatment of adenomyosis, such as selective progesterone receptor modulators, aromatase inhibitors, GnRH-antagonists, danazol, valproic acid, modulation of prolactin and/or oxytocin, and antiplatelet therapy. However, the investigations of these options have been confined to their effect on the symptoms of adenomyosis; studies concerning their impact on reproductive outcomes are still missing [67,68].

Adolescents (aged 12–20 years) diagnosed with adenomyosis but with no current desire for children are in a special situation [37]. The question arises as to whether one could improve or preserve fertility a priori, given the presence of adenomyosis. The treatment recommendation depends on the symptoms. In cases of dysmenorrhea or a bleeding disorder, hormonal treatment with a combined pill, progestogen only or LNG-IUS appears to be advisable. In cases of asymptomatic patients or those with mild symptoms, unequivocal treatment recommendations cannot be made due to the lack of data on subsequent fertility outcomes.

In recent years, several risk factors have been identified for the development of adenomyosis. Estrogen exposure appears to be a primary risk factor [69]. By implication, hormonal treatment that reduces estrogen levels by inhibiting ovulation (such as combined oral contraceptives or progestogen mono) could counteract the progression of adenomyosis and thus increase fertility rates in the future. Nevertheless, clear therapy recommendations can only be issued after further studies have been performed on these patients.

### 7.2. Surgical Treatment Options

In surgical treatment, a distinction is made between diffuse adenomyosis and adenomyoma, i.e., localized or focal adenomyosis. An adenomyoma can usually be excised in toto without difficulty, although identification of the layers is usually more difficult than in the case of a myoma. Surgical excision may also be useful in cases of diffuse adenomyosis. Several techniques have been described, although none appears to be superior to the others. Techniques used for adenomyoma (focal adenomyosis) and diffuse adenomyosis are shown in Table 4. The classic technique in case of focal adenomyosis means an open or laparoscopic complete adenomyomectomy and includes the same steps performed in a myomectomy. The classic technique in case of diffuse adenomyosis starts with a vertical or transverse incision in the middle of the uterine wall, recognition and resection of all macroscopic lesions (cytoreductive surgery) and a final wound closure in at least two layers, with care taken not to leave any uterine defect behind [70]. The disadvantage of surgery in diffuse adenomyosis is that the rate of uterine rupture in a future pregnancy appears to be 4–6%, which is higher uterine rupture rates after myomectomy or cesarean section [70,71]. Other pregnancy complications, such as placental disorders, have also been reported. Women undergoing resection should be educated about the need for cesarean section on a labor-free uterus [70].

In a review comprising 64 studies and 1049 patients, Grimbizis et al. noted a reduction of dysmenorrhea and menorrhagia control in 82% and 68.8%, respectively, after complete excision. In addition, the study population achieved a high pregnancy rate of 60.5%. Even after partial excision, a reduction of dysmenorrhea was noted in 81.8%, control of menorrhagia in 50.0%, and pregnancies were achieved in 46.9% of cases [72].

Another systematic review comprising 18 studies and 1396 infertile women with focal and diffuse adenomyosis (AD) analyzed reproductive outcomes after uterine-sparing surgery [73]. Patients with focal AD achieved mean pregnancy and miscarriage rates of 52.7% and 21.1% respectively, whereas patients with diffuse AD had mean pregnancy and miscarriage achieved rates of 34.1% and 21.7%, respectively. Uterine rupture and preterm birth were observed in 6.8% and 4.5% of pregnant patients with diffuse AD versus 0% and 10.9% of patients with focal AD, respectively. No significant differences were noticed between natural conception compared with assisted reproductive technology (ART). Overall, patients with focal AD appear to achieve higher pregnancy rates after conservative surgery compared with diffuse AD, whereas a higher incidence of uterine rupture was reported after surgery for diffuse AD [73]. The indication for surgical resection of adenomyosis must be discussed individually with each patient. Surgery should be recommended especially in cases of younger infertile women who have failed medical management. Sufficient contraception must be discussed with the patient postoperatively for at least 6–12 months before the patient seeks to conceive again. In addition, the patient must have an adequate chance to conceive spontaneously due to the age factor. A demonstration of statistical data from the German IVF Registry (DIR) regarding pregnancy and miscarriage rates may be helpful in this regard.

Surgery can be also recommended in older women with infertility despite ART, and those with a history of recurrent pregnancy loss or implantation failure. There are no rigid age limits that precisely define young and old patients. Rather, the procedure should be discussed individually with the respective patient, taking into account the time of the unfulfilled desire for a child, the age of the patient and the respective egg reserve.

Hysteroscopic resection is indicated in patients with adenomyosis limited to the endo-myometrial junction or adenomoysis foci close to the uterine cavity, and is frequently performed with ultrasound guidance for better detection. Good results have been reported in cases of abnormal uterine bleeding and dysmenorrhea, but endo-myometrial resection is contraindicated in patients who desire pregnancy because it causes destruction of the endometrium together with the JZ. This may lead to higher rates of miscarriage, preterm labor, and placenta-related complications [21,74].

### 7.3. Other Methods

High-intensity focused ultrasound (HIFU) or focused ultrasound surgery is a non-invasive local thermal ablation technique. The ultrasound beams penetrate the tissue through the acoustic pathway and are then focused on the target tumor within the body. When the temperature increases to 65 °C, coagulative necrosis occurs in the tumor [75,76]. The treatment is performed under ultrasound or MRI guidance and can be used in cases of focal as well as diffuse adenomyosis. HIFU provides effective and long-term pain and bleeding control, and its application is safe. Patients must be carefully selected to avoid severe complications, such as skin burns (0.2%) or intestinal injuries (0.02%) [76]. The body of data concerning the effect of HIFU in infertile patients is small. Zhou et al. performed a follow-up analysis of 68 HIFU-treated adenomyosis patients who wished to conceive. Of 68 patients, 54 conceived at a median of 10 months (range, 1–31 months) after treatment, and 21 of them delivered [77]. Another study with a similar sample size showed comparable results: 52 adenomyosis patients were treated with HIFU from 2011 to 2016 (Chongqing, China). A total of 20 patients conceived at a median of 8.75 months after HIFU, and 11 delivered at term [76]. Both study groups observed no uterine rupture during pregnancy or delivery. Although the sample sizes were small, the above mentioned results have shown that HIFU treatment does not increase the risk of complications during gestation and delivery. In addition, due to the absence of scar tissue on the uterine wall, patients may attempt to conceive much sooner than they would after surgical treatment. Moreover, the risk of uterine rupture during pregnancy or delivery is lower than after surgery [21].

Hysteroscopic endometrial ablation is frequently performed together with hysteroscopic resection and refers to the coagulation of adenomyosis cysts and crypts [78]. Hysteroscopic ablation can be conducted with yttrium aluminum garnet (YAG) laser, rollerball resection, thermal balloon ablation, cryoablation, circulated hot fluid ablation, microwave ablation, bipolar radiofrequency ablation, or electrocoagulation [79]. Endomyometrial ablation is effective for lesions deeper than the endometrial-myometrial junction, whereas the efficacy of hysteroscopic ablation is limited to foci at a depth of 2–3 mm. Pregnancies have been reported after endometrial ablation, but there is little data about their outcomes. Kohn et al. performed a systematic review of 274 pregnancies from 99 sources, of which 78 were case reports. Women aged 26–50 years (mean 37.5 +/− 5 years) conceived at a median of 1.5 years after hysteroscopic ablation [80]; 85% of pregnancies ended in miscarriage, induced abortion or ectopic pregnancy. Pregnancies that continued had high rates of preterm delivery, caesarean delivery, caesarean hysterectomy, and adherent placenta [80]. Many case reports described a higher risk of preterm premature rupture of membranes, intrauterine growth restriction, intrauterine fetal death, and uterine rupture [80]. Due to the high rate of pregnancy complications as a result of damage to the endometrium, hysteroscopic ablation should not be performed in patients who desire pregnancy.

Uterine artery embolization (UAE) is a means of achieving necrosis of adenomyotic lesions with the aid of transarterial catheters. Vascular access is gained through a femoral or radial artery puncture, and the arteriography is followed under fluoroscopic guidance. Embolization is usually performed using permanent particulate agents of various sizes. The technique used for UAE in adenomyosis is similar to that used for fibroids. Over the last 15 years, UAE has been employed quite extensively for the treatment of symptomatic adenomyosis, and has yielded favorable short- and long-term outcomes [78,81]. Mohan et al. performed a systematic review analyzing the outcome of fertility after UAE. Low-level evidence from these studies suggests that pregnancy rates after UAE are comparable to age-adjusted rates in the general population [82]. Although pregnancy complication rates were comparable to those in patients with untreated fibroid tumors, a few studies have reported higher rates of miscarriage after UAE [82].

In contrast, the current American College of Obstetrics and Gynecology and Society of Interventional Radiology guidelines still mention the desire for future fertility as a relative contraindication to UAE. This recommendation is corroborated by a number of studies concerning reproductive outcomes after UAE in patients with fibroids. Compared to surgical fibroid enucleation, UAE resulted in lower pregnancy rates and higher miscarriage rates [83,84]. Further randomized studies will be needed to make a clear recommendation for these patients [78].

## 8. Conclusions

Adenomyosis is a common gynecological disorder, affecting women of reproductive age. Higher rates of implantation failure, recurrent pregnancy loss, and preterm birth have been associated with this diagnosis. The absence of an exact image classification system has limited our ability to assess adenomyosis in terms of its extent and severity. Assessing the severity of adenomyosis would permit precise therapy recommendations. A number of therapy approaches exist. The studies have primarily focused on the alleviation of symptoms, such as bleeding disorders or dysmenorrhea. Randomized controlled trials evaluating the impact of adenomyosis on reproductive outcomes are still missing. Surgery alleviates the symptoms and has been successful in regard to fertility outcomes, but may increase the risk of uterine rupture. HIFU appears to be a safe treatment option for patients who wish to conceive. However, we need randomized clinical trials comparing HIFU with other treatment options. Other approaches for the treatment of adenomyosis in infertility patients—such as hysteroscopic ablation or UAE—cannot be recommended without restriction at the present time.

Pretreatment with GnRH analogs prior to natural conception or as an ultra-long protocol before ART is associated with a positive effect on reproductive outcomes. Surgery should be offered to symptomatic women with repeated implantation failure after ART. Any unequivocal evidence-based recommendation is hindered by the limited body of existing data. Possibly, new pharmacological approaches such as selective progesterone receptor modulators, AIs or GnRH antagonists may provide benefits in terms of reproductive outcomes in women with adenomyosis and infertility.

## Figures and Tables

**Table 1:** Clinical symptoms, concomitant diseases and their frequency in adenomyosis [10,15,16].

Clinical Symptoms	Frequency (%)
Chronic lower abdominal pain	77
Hypermenorrhea	40–50
Dysmenorrhea	15–30
Asymptomatic	30
Dyspareunia	7
Infertility	7–28
**Concomitant diseases**	
Fibroids	50
Endometriosis	11
Endometrial polyps	7

**Table 2:** Ultrasound features of diffuse and focal adenomyosis [21,24,26].

2D US Feature	Diffuse Adenomyosis	Focal Adenomyosis
Serosal contour of the uterus	The uterus is frequently enlarged in its en tirety	Usually regular
Definition of the lesion	Indistinct	Usually well-defined in cases of cystic or hyperecho genic lesions surrounded by a normal myometrium
Symmetry of uter ine walls	Wall difference in favor of the anterior or pos terior wall (pseudo-widening sign)	Often symmetric
Shape	Indistinct	Indistinct, oval in cases of cystic lesions
Contour	Indistinct	Irregular or indistinct
Shadowing	Fan-shaped shadowing Linear hypoechoic striation	Rarely fan-shaped shadowing or linear hypoechoic striation
Echogenicity	Diffuse Presence of intramyometrial diffuse areas of: mixed echogenicity Small cyst echogenic islands echogenic subendometrial lines	Focal, surrounded by normal myometrium Presence of intramyometrial focal small areas of: mixed echogenicity small and large cyst echogenic islands echogenic subendometrial lines or buds
Vascularity	Translesional flow Diffuse minimal or few vessels	Diffuse minimal, sporadic vessels
Endometrial rim	Irregular and distorted	Often regular or imprinted by subendometrial focal lesion

Abbreviations: US = ultrasound.

**Table 3:** Treatment options (including pretreatment) of adenomyosis in infertility patients.

Pharmacological	Recommendation
NSAIDs	ambiguous
Combined oral contraceptives (mostly long cycle)	ambiguous
Progestogen mono (e.g., Desogestrel, Dienogest)	+/(−)
Levonorgestrel IUS (e.g., Mirena^®^, Jaydess^®^, Kyleena^®^)	+
GnRH analogs (e.g., leuprorelin, tritorelin, buserelin)	+
New approaches (selective progesterone receptor modulators, AIs, GnRH antagonists, danazol, valproic acid, modulation of prolactin and/or oxytocin and antiplatelet therapy	ambiguous
**Surgical**	
Complete resection of adenomyoma/ localized adenomyosis (laparo scopic)	+
Cytoreductive surgery of diffuse adenomyosis (laparoscopy, better laparotomy)	(+)
Hysteroscopic resection	-
**Other methods**	
High-intensity focused ultrasound	+
Hysteroscopic endometrial ablation	-
Uterine artery embolization	-

The small number of existing studies with limited sample sizes make it difficult to issue clear recommendations for adenomyosis and the success of reproduction. Ambiguous: missing data, +/(-): most of the studies show positive effects, a few no benefit, +: positive effect, (+): questionable positive effect, -: not recommended. Abbreviations: NSAID: nonsteroidal anti-inflammatory drugs; IUS: intrauterine system; GnRH: gonadotropin-releasing hormone, AI: aromatase inhibitor.

**Table 4:** Surgical techniques for focal and diffuse adenomyosis.

Characteristics	Type I	Type II
	Focal or localized adenomyosis	Diffuse adenomyosis
Extent of excision	Complete (if possible)	Cytoreductive surgery
Route of surgery	Laparoscopic or via laparotomy	Laparoscopic or via laparotomy
Techniques	Classic technique (adenomyomectomy)	Classic technique
	U-shaped technique	Transverse H-shaped incision
	Overlapping flaps	Wedge resection of the uterus
	Triple-flap method	Asymmetric dissection of the uterus

## Data Availability

The datasets analyzed for the current study are available from the corresponding author on reasonable request.

## Abbreviations

TVS	transvaginal ultrasound
MRI	magnetic resonance imaging
JZ	junctional zone
ART	assisted reproductive technologies
IVF	in vitro fertilization
NSAIDs	nonsteroidal anti-inflammatory drugs
IUS	intrauterine system
GnRH	gonadotropin releasing hormone
AI	aromatase inhibitor
OC	Combined oral contraceptive
POP	Progestin-only pill
DNG	Dienogest
LNG-IUS	Levonorgestrel intrauterine system
AD	adenomyosis
HIFU	High intensity focused ultrasound
YAG	yttrium aluminum garnet
UAE	Uterine artery embolization
